# Maternal Vitamin D, Folate, and Polyunsaturated Fatty Acid Status and Bacterial Vaginosis during Pregnancy

**DOI:** 10.1155/2011/216217

**Published:** 2011-12-08

**Authors:** Anne L. Dunlop, Robert N. Taylor, Vin Tangpricha, Stephen Fortunato, Ramkumar Menon

**Affiliations:** ^1^Department of Family & Preventive Medicine, Emory University School of Medicine, Atlanta, GA 30322, USA; ^2^Department of Gynecology and Obstetrics, Emory University School of Medicine, Atlanta, GA 30322, USA; ^3^Department of Medicine, Emory University School of Medicine, Atlanta, GA 30322, USA; ^4^Perinatal Research Center, Women's Health Research and Education Foundation, Nashville, TN 37203-1538, USA; ^5^Division of Maternal-Fetal Medicine Perinatal Research, Department of Obstetrics & Gynecology, The University of Texas Medical Branch at Galveston, Galveston, TX 77555-1062, USA

## Abstract

*Objective*. To investigate associations among serum 25-hydroxy-vitamin D (25-OH-D), folate, omega-6/omega-3 fatty acid ratio and bacterial vaginosis (BV) during pregnancy. *Methods*. Biospecimens and data were derived from a random sample (*N* = 160) of women from the Nashville Birth Cohort. We compared mean plasma nutrient concentrations for women with and without BV during pregnancy (based on Nugent score ≥7) and assessed the odds of BV for those with 25-OH-D <12 ng/mL, folate <5 ug/L, and omega-6/omega-3 ratio >15. *Results*. The mean plasma 25-OH-D was significantly lower among women with BV during pregnancy (18.00±8.14 ng/mL versus 24.34±11.97 ng/mL, *P* = 0.044). The adjusted odds of BV were significantly increased among pregnant women with 25-OH-D <12 ng/mL (aOR 5.11, 95% CI: 1.19–21.97) and folate <5 ug/L (aOR 7.06, 95% CI: 1.07–54.05). *Conclusion*. Vitamin D and folate deficiencies were strongly associated with BV (Nugent score ≥7) during pregnancy.

## 1. Introduction

Bacterial vaginosis (BV), a polymicrobial clinical syndrome resulting from replacement of the normal vaginal flora with high concentrations of anaerobic bacteria [1], commonly affects women of reproductive age in the United States [2, 3]. BV is associated with adverse gynecologic and obstetrical outcomes. Among nonpregnant women, BV is associated with endometritis [4], postabortion endometritis [5], and nonchlamydial-nongonococcal pelvic inflammatory disease [6]. BV also increases a woman's risk of acquiring and transmitting HIV infection [7–13] and is associated with increased prevalence of herpes simplex virus 1 and 2 [14] as well as Neisseria gonorrhoeae and Chlamydia trachomatis infection [15]. Pregnant women with BV are at increased risk of premature rupture of the fetal membranes [16], chorioamnionitis and intra-amniotic infection [17], postcesarean delivery endometritis [18], postpartum complications of the infant [19], and spontaneous preterm birth [20–24]. Randomized controlled trials among women who had previously experienced a spontaneous preterm birth show a reduction in the recurrence of preterm birth following treatment of BV [25]. It is estimated that the significantly higher rates of BV among black women may account for up to one-third of the black-white racial disparity in preterm birth in the United States [26].

The etiology of and risk factors for BV are not completely understood. A recent cohort study found that black race, cigarette smoking, vaginal intercourse, receptive anal intercourse before vaginal intercourse, sex with an uncircumcised male partner, and the presence of herpes simplex virus 2 antibodies are independently associated with the acquisition of BV [27]. BV is three times more prevalent among black compared with white women [2], but factors such as socioeconomic status, more frequent douching, and sexual intercourse, and other recognized risk factors for BV do not account for the racial disparity [2, 28].

It is theorized that factors that impair the immune system, including micronutrient deficiencies, may increase susceptibility to BV. One US cohort study has examined the association between maternal vitamin D status and BV in pregnancy, finding a dose-response relationship between the mean unadjusted serum 25-OH-D concentration and the prevalence of BV per Gram stain of vaginal smears obtained in early pregnancy [29]. National Health and Nutrition Examination Survey (NHANES) data from 2001–2004 reveal that vitamin D deficiency is associated with BV in pregnant, but not nonpregnant, women (aOR 2.87, 95% CI: 1.13–7.28) [30]. Another US cohort study among nonpregnant women found an association between BV and the high total dietary intake of fat (aOR 1.5, 95% CI: 1.1–2.4) and the low dietary intake of folate (aOR 0.4, 95% CI: 0.2–0.8), vitamin E (aOR 0.4, 95% CI: 0.2–0.8), and calcium (aOR 0.4, 95% CI: 0.3–0.7), whereas no relationship with dietary vitamin D intake was observed [31]. A cohort study of low-income, pregnant African-American women found no association between BV and dietary intake of protein and vitamins A and C [32]. A case-control study of well-nourished pregnant Belgian women found a significantly increased risk of BV among women with subclinical iron deficiency [33].

Existing studies of micronutrient status and BV during pregnancy have concluded that replication of these findings in other study populations, especially those that are racially heterogeneous, is important for further investigating the potential relationship between maternal micronutrient status and BV during pregnancy. The purpose of this study was to explore whether maternal serum concentrations of 25-OH-D, folate, and omega-6/omega-3 polyunsaturated fatty acids were associated with the diagnosis of BV (Nugent score ≥7) during the pregnancy.

## 2. Materials and Methods

### 2.1. Overview

The subjects for this study were drawn from women enrolled in the Nashville Birth Cohort as part of an on-going genetic study of racial disparities in spontaneous preterm birth at Centennial Women's Hospital, Nashville, Tenn, USA, a tertiary care hospital that receives referrals and transfers of high-risk patients from an area that encompasses a 100 mile radius around Nashville [34]. This study involved the biochemical assessment of vitamin D, folate, and polyunsaturated fatty acid status from stored EDTA plasma samples collected during the delivery admission and comparison of vitamin D, folate, and polyunsaturated fatty acid nutriture for women with and without a diagnosis of BV during the pregnancy.

The study was approved by the Western Institutional Review Board. All patient participants provided written informed consent at the time of enrollment; the consent form specifically sought permission to store biosamples for future testing and research. The Emory University Institutional Review Board also approved the study protocol (no. 31456).

### 2.2. Study Sample

Biospecimens and medical record data for this study (*N* = 160) reflect a subsample of the 1,547 total women enrolled into the Nashville Birth Cohort during 2003–2006. Using a stratified random sampling strategy based upon computer-generated random number selection, 80 women (40 non-Hispanic black and 40 non-Hispanic white) with spontaneous preterm birth and 80 women (40 non-Hispanic black and 40 non-Hispanic white) with term birth were selected from among those for whom sufficient stored plasma samples were available for planned biochemical analyses. This project examined samples from 160 women as we had sufficient funding to perform the biochemical assessments for this number of subjects.

Criteria for enrollment into the Nashville Birth Cohort included being a woman ≥18 years at the time of the delivery admission who was pregnant, nulli- or primiparous upon enrollment with a singleton infant without anomalies. Women with multiple gestations, preeclampsia, placenta previa, fetal anomalies, medical/surgical complications of pregnancy, and drug or alcohol abuse were excluded. Preeclampsia was defined as a blood pressure on two separate readings taken at least 4 hours apart of 140/90 or more *and* ≥300 mg of protein in a 24-hour urine sample (proteinuria). Subjects who had any surgical procedures during pregnancy or who were treated for preterm labor or for suspected intraamniotic infection and delivered at term were excluded.

### 2.3. Clinical Data

Clinical data obtained from the Nashville Birth Cohort were used for this study, including the following.

**(1) Gestational Age**

The gestational age at delivery was ascertained by review of the prenatal records. Both the last menstrual period and ultrasound before the 20th week of gestation were used to assign gestational age. If the last menstrual period and ultrasound results were discordant, the ultrasound result was used, according to accepted clinical criteria.

**(2) Obstetrical History and Pregnancy Outcome**

Information related to the number of prior pregnancies and births and the outcomes of pregnancies and births was ascertained via review of the prenatal record (which included an obstetrical interview conducted at the first prenatal visit). Using the gestational age of the index pregnancy, the deliveries were dichotomized as preterm (22-0/7 through 36-6/7 weeks) or term (≥37 weeks).

**(3) Maternal Race/Ethnicity**

Maternal race was self-identified based on the race/ethnicity of their own parents and grandparents. Those who identified as being of Hispanic ethnicity, as well as those of mixed race, and those who were unsure of the race/ethnicity of their parents and grandparents were excluded from this study. Those who identified each of their parents and grandparents as black were regarded as black for this study, and similarly for whites as we have reported previously [34].

**(4) Body Mass Index (BMI)**

Prepregnancy BMI was calculated from measured height at the first prenatal visit and patients' report of their Prepregnancy weight at the first prenatal visit. BMI was categorized according to accepted definitions (obesity ≥30 kg/m^2^, overweight 25–29.99 kg/m^2^, healthy weight 18.5–24.99 kg/m^2^, and underweight <18.5 kg/m^2^).

**(5) Bacterial Vaginosis**

Women enrolled into the cohort at Centennial Women's Hospital received prenatal care through obstetrical care providers who follow a clinical protocol that involves performing a pelvic speculum examination at the initial obstetrical visit to ascertain for the presence of BV according to Nugent's criteria. Vaginal swabs from the pelvic examination were transported to the clinical pathology laboratory at Centennial Women's Hospital for Gram staining and determination of BV according to Nugent's criteria, with a score ≥7 considered positive [35].

### 2.4. Biological Specimens and Nutrient Analyses

Blood samples were obtained at the time of the delivery admission for all consented subjects. Nonfasting venous blood drawn routinely as part of the delivery admission was obtained, and an extra aliquot of EDTA plasma was centrifuged and stored at −80°C degrees. Thawed EDTA plasma was analyzed for the following nutrients, and measurements were categorized as described below. All assays were performed by technicians without knowledge of case-control status.

**(1) Vitamin D**

Plasma 25-OH-D concentration, the major circulating form of vitamin D, was assayed using a commercially available ELISA kit (Immunodiagnostic Systems, Fountain Hills, AZ), the range of detection for which is 2–120 ng/mL. Analyses were performed at the Vitamin D and Bone Research Laboratory at Emory University School of Medicine, which participates in the vitamin D external quality assessment scheme (http://www.deqas.org/) and in the National Institutes of Health/National Institute of Standards and Technology (NIH/NIST) Vitamin D Metabolites Quality Assurance Program (VitDQAP) to ensure accuracy of the 25-OH-D measurements. In accordance with a recent review of data by a committee of the Institute of Medicine, the following serum concentrations of 25-OH-D were considered in the analyses: <12 ng/mL (vitamin D deficient), 12–20 ng/mL (vitamin D insufficient), ≥20 ng/mL (referrent category, considered adequate for bone and overall health in healthy individuals) [36].

**(2) Folate**

Plasma folate concentration was measured by competitive-binding chemiluminescent radioimmunoassay using competitive displacement of 125-I folic acid from intrinsic factor and folate binding proteins immobilized on microcrystalline cellulose. Analyses were performed by Emory Healthcare laboratory, an accredited clinical and research laboratory using a Beckman Coulter DX1800 (Beckman Coultern, Inc., Fullerton, Calif). Folate deficiency was defined as a measured serum concentration <5 ug/L [37].

**(3) Omega-6 and Omega-3 Fatty Acids**

Fatty acid analyses were performed in the Lipoprotein Analysis Laboratory, Wake Forest University School of Medicine. In brief, the total lipid fraction was extracted from 100 uL of plasma, and the phospholipid fraction was isolated by liquid chromatography on silica gel plates. Fatty acid methyl esters were prepared by transesterification involving saponification with 0.5 NaOH in methanol and methylation using 14% BF_3_ in methanol followed by extraction in hexane. The fatty acid methyl esters were separated via capillary column gas chromatography and identified by flame ionization. Retention times were determined with mixed fatty acid methyl ester standards from NuChek Prep (Elysian, Minn), and quantification by comparison with the peak areas of the internal standards provided absolute concentrations [38]. Polyunsaturated fatty acids were expressed as a ratio of total omega-6 to omega-3 fatty acids. Presently, there is not a defined cut-point for an excessive omega-6/omega-3 fatty acid ratio; in this study, the highest tertile was a ratio of omega-6/omega-3 >15, and this was used to categorize the data.

### 2.5. Data Analysis

We compared demographic and clinical characteristics of women with and without BV using Student's *t*-test for continuous measures and Pearson's Chi-square or Fisher's exact test for categorical measures. We compared the mean concentrations of plasma 25-OH-D and folate and the mean ratio of total omega-6/omega-3 fatty acids for women with and without BV using Student's *t*-test. We compared the proportion of women with and without BV who had plasma 25-OH-D <12 and <20 ng/mL, folate <5 ug/L, and total omega-6/omega-3 fatty acid ratio >15 using Pearson's Chi-square test. We stratified the comparison of BV × black versus white race, obese versus nonobese status and nutrient status, assessing for biological interaction using the Breslow-Day test statistic. We used multivariate logistic regression to assess the independent and interactive effects of the nutrient measures in separate models that included covariates noted to be linked with BV in previous research, including age, race, obesity, health insurance status, marital status, and smoking status. All hypothesis testing and reported probability values were two-tailed and conducted at the *α* = 0.05 level. Statistical analyses were conducted using SPSS 17.0 (SPSS, Inc.).

## 3. Results

Among the study sample, 14/146 (8.8%) of women were diagnosed with BV (Nugent Score ≥7) during the pregnancy. Characteristics of women with and without BV during the pregnancy are given in Table 1. There was no significant difference in the mean maternal age, years of education, BMI, or gestational age at delivery among women with and without BV during the pregnancy. Of the nutrients under study, there was a significant difference in the mean plasma concentration of 25-OH-D for women with and without BV during the pregnancy (18.00 ± 8.14 ng/mL versus 24.34 ± 11.97 ng/mL, *P* = 0.044). The mean plasma concentration of folate and the mean ratio of omega-6/omega-3 polyunsaturated fatty acids were greater among those with BV compared to those without BV during the pregnancy, but differences were not significant (Table 1).

The proportion of women with particular characteristics and who had measured nutrient values outside the cut-off values of interest, along with the crude and adjusted odds of BV for women with these characteristics, is given in Table 2. In neither univariate or multivariate analysis was black race, being a teenager, obesity, or medicaid status associated with BV. In multivariate analysis, the odds of BV were significantly greater among smokers (aOR 3.16, 95% CI: 1.08–10.21). In both univariate and multivariate analysis, 25-OH-D <12 ng/mL and folate <5 ug/L increased the odds of BV during pregnancy. Conversely, omega-6/omega-3 ratio >15 was not significantly associated with BV in univariate or multivariate analysis. Stratified analyses revealed no important biological interactions for the relationship between BV and the nutrients of interest according to race or obesity status.

## 4. Discussion

In this study, we observed a significantly lower mean plasma concentration of 25-OH-D among women diagnosed versus not diagnosed with BV during pregnancy as well as a significant association between maternal serum 25-OH-D <12 ng/mL and folate <5 ug/mL and the occurrence of BV during pregnancy. In the case of vitamin D deficiency, our findings are consistent with a recent cohort of pregnant women, which found a dose-response relationship between 25-OH-D and the prevalence of BV during pregnancy [29], and a nationally representative cross-sectional study of women in the United States, which found that 25-OH-D <30 ng/mL significantly increased the odds of BV during pregnancy (aOR 2.87, 95% CI: 1.13–7.28) [30]. However, it is notable that, in our study, the odds of BV were only significantly greater for women with a serum 25-OH-D <12 ng/mL and not for those with serum 25-OH-D <20 ng/mL. In the case of folate deficiency, our findings are consistent with a prior cohort study finding of a relationship between low-dietary folate intake and BV, although that study was among nonpregnant women and examined dietary intake as opposed to plasma folate concentration [31].

Existing research supports that micronutrient status is linked with susceptibility to infection, lending biologic plausibility to the observed associations between maternal nutrient status and BV during pregnancy. The active form of vitamin D (1,25-dihydroxyvitamin D_3_) is a key modulator of the immune response, and vitamin D is known to be a potent regulator of placental immunity, stimulating antimicrobial responses while suppressing inflammation [39]. There is emerging evidence that vitamin D regulates a key antimicrobial peptide with broad antimicrobial properties against gram-negative and gram-positive bacteria [40]. Adequate vitamin D status may offer protection against BV by induction of cathelicidin [41]. Low serum folate is associated with impaired T cell and neutrophil function, and deficiency of folate is associated with an increased risk of bacteriuria in pregnancy [42–44].

Studies in the US demonstrate a higher prevalence of deficiencies of vitamin D and folate among black compared with white women. Circulating levels of 25-OH-D are a direct reflection of total body vitamin D status, which depends upon access to vitamin D either through exposure to sunlight or dietary intake. NHANES data from 1988–1994 reveal that 42% of non-Hispanic black women of child-bearing age had 25-OH-D levels lower than 37.5 nmol/L (15 ng/mL) compared with only 4% of non-Hispanic white women [45]. Also, suboptimal vitamin D status, defined as 25-OH-D <75 nmol/L (30 ng/mL), is more prevalent among non-Hispanic black compared with non-Hispanic white women (74–95% versus 46–62%) [46]. According to 2001-2002 NHANES data, non-Hispanic black women have lower folate intake from both food and supplement sources. Whereas approximately 40% of non-Hispanic white women consume 400 ug of folic daily, only 5% of non-Hispanic black women achieve 400 ug of folic acid daily from fortified foods and supplements. The prefortification mean red blood cell folate was lower for non-Hispanic black compared with non-Hispanic white women, and fortification produced a smaller shift in mean red blood cell folate levels for black compared with white women [47].

Existing studies also clearly demonstrate significantly higher rates of BV among black compared with white women and estimate that the significantly higher rates of BV among black women may account for up to one-third of the black-white racial disparity in preterm births [26]. Thus, in addition to the myriad other health reasons for addressing the poor underlying vitamin D and folate status of US women [48, 49], decreasing the susceptibility of women of reproductive age to BV may provide further rationale.

Strengths of this study include its use of the microbiologic gold standard for the determination of BV and its use of biochemical assessment of nutrient status. Another strength is that pregnant women who entered the birth cohort were assessed for BV upon their initial obstetrical visit regardless of their symptom status, thereby increasing the generalizability of our study findings.

The present study is not without limitations. First, the measurement of serum nutrient status occurred upon admission to the labor and delivery unit, thereby limiting our ability to evaluate the potential importance of periconception and early pregnancy nutritional status and BV. While this may have contributed to our findings, previous longitudinal research documents that maternal fatty acid values remain constant throughout pregnancy [50] and 25-OH-D concentrations do not differ significantly throughout gestation [51, 52]. Second, because subjects were enrolled during their delivery admission with spontaneous onset of labor, we were restricted to the use of Nonfasting samples. The use of Nonfasting specimens could have influenced our findings; however, a previous study that assessed prenatal dietary intake and Nonfasting plasma fatty acids found high correlations between fasting and postprandial plasma lipid measures (ranging from 0.90 to 0.99, *P* < 0.001) [53]. Another limitation is that, due to having access to only stored plasma, we were able to measure circulating folate and fatty acids but not intracellular erythrocyte folate and fatty acid concentrations. Red blood cell measures of these nutrients provide a more stable and reliable measure of nutrient status over several months [54, 55]. However, previous research demonstrates very high correlations between plasma and red blood cell folate and fatty acids [56, 57]. A final limitation is that a relatively small proportion of women in the study sample met Nugent's criteria for the diagnosis of BV, thereby limiting the power of the study for detecting the assessing of the relationship of the nutrients with BV. It is possible that, had the study protocol called for repeat testing for BV throughout the pregnancy, whether women were symptomatic or not, that a higher proportion of women in the sample may have met criteria for the diagnosis of BV in pregnancy.

## 5. Conclusions

This case-control study found a significant association between maternal plasma 25-hydroxy-vitamin D <12 ng/mL and folate <5 ug/mL and BV during pregnancy. Given the growing evidence that pregnant women with BV are at increased risk of a number of adverse pregnancy outcomes, including spontaneous preterm birth, prospective research studies that assess nutrient status and BV prevalence early in pregnancy, identify incident cases of BV during the pregnancy, and assess the role of supplementation on BV prevalence and incident cases as well as on important pregnancy outcomes are needed to refine our understanding of the role of micronutrients in infectious and pregnancy-related outcomes.

## Figures and Tables

**Table 1:** Characteristics of women with and without bacterial vaginosis during pregnancy.

Characteristic	Bacterial vaginosis during pregnancy (*n* = 14)	No bacterial vaginosis during pregnancy (*n* = 146)	*P* value
Maternal age—mean years (sd)	24.14 ± 6.04	26.13 ± 6.06	0.946
Education—mean years (sd)	13.57 ± 2.56	13.80 ± 2.60	0.904
BMI—kg/m^2^ (sd)	27.25 ± 8.87	25.99 ± 6.63	0.135
Gestational age at delivery—weeks (sd)	34.90 ± 3.37	36.62 ± 2.99	0.086
25-OH-D (ng/mL)—mean (sd)	18.00 ± 8.14	24.34 ± 11.97	**0.044**
Folate (ug/L)—mean (sd)	7.9 ± 5.1	10.7 ± 4.6	0.086
Omega-6/omega-3 ratio—mean (sd)	13.8 ± 3.4	13.0 ± 2.5	0.265

**Table 2:** Crude and adjusted odds for bacterial vaginosis during pregnancy according to maternal characteristics.

Characteristic	Bacterial vaginosis (*n* = 14)	No bacterial vaginosis (*n* = 146)	cOR (95% CI)	aOR (95% CI)*
Black race (%)	8 (57.1%)	72 (49.3%)	1.37 (0.45, 4.15)	1.17 (0.36, 3.76)
Teenager (%)	4 (28.6%)	19 (13.0%)	2.67 (0.76, 9.39)	2.27 (0.53, 9.79)
Smoker (%)	6 (42.9%)	31 (21.2%)	2.78 (0.92, 8.61)	**3.16 (1.08, 10.21)**
Obese (%)	4 (30.8%)	40 (27.8%)	1.16 (0.34, 4.01)	1.33 (0.36, 4.92)
Delivery <37 weeks (%)	10 (71.4%)	70 (47.9%)	2.71 (0.87, 9.05)	1.96 (0.64, 7.10)
TennCare** (%)	8 (57.1%)	74 (50.7%)	1.16 (0.78, 3.85)	1.79 (0.31, 10.32)
Folate <5 ug/L—*n* (%)	3 (21.4%)	4 (4.0%)	** 6.55 (1.29, 33.13)**	**7.06 (1.07, 54.05)**
25-OH-D <12 ng/mL—*n* (%)	5 (35.7%)	10 (6.8%)	** 7.58 (2.13, 27.03)**	**5.11 (1.19, 21.97)**
25-OH-D <20 ng/mL—*n* (%)	7 (50%)	62 (42.5%)	1.4 (0.5, 4.1)	1.2 (0.39, 3.85)
Omega-6/omega-3 > 15—*n* (%)	12 (85.7%)	92 (65.2%)	3.19 (0.79, 14.93)	3.98 (0.78, 13.7)

*The multivariate model included race, age, smoking status, body mass index status, gestational age at delivery, and payor source.

**Tennessee's Medicaid Managed Care Program that provides health insurance coverage for low-income, pregnant women.
